# Toward Sustainable Management of *Spodoptera frugiperda* (Lepidoptera: Noctuidae): A Review on the Role of Endophytic Fungi in Crop Protection

**DOI:** 10.3390/plants15152375

**Published:** 2026-08-03

**Authors:** Nongamanégré Kouanda, Ibtissem Ben Fekih, Marcellin C. Cokola, Anne-Lise Hanstson, Rudy Caparros Megido, Frank Delvigne, Athanase Badolo, Frédéric Francis

**Affiliations:** 1 Functional and Evolutionary Entomology, Gembloux Agro-Bio Tech, University of Liège, 5030 Gembloux, Belgium; marcellin92cokolacuma@gmail.com (M.C.C.); r.caparros@uliege.be (R.C.M.); frederic.francis@uliege.be (F.F.); 2 Fundamental and Applied Entomology Laboratory, Unité de Formation et de Recherche en Sciences de la Vie et de la Terre (UFR SVT), Université Joseph Ki-Zerbo, Ouagadougou 03 BP 7021, Burkina Faso; a.badolo@gmail.com; 3 Department of Plant Production, Faculty of Agriculture and Environmental Sciences, Université Evangélique en Afrique, Bukavu P.O. Box 3323, Democratic Republic of the Congo; 4 Chemical and Biochemical Engineering, Polytechnic Faculty, Université de Mons, Place du Parc 20, 7000 Mons, Belgium; anne-lise.hantson@umons.ac.be; 5 Microbial Processes and Interactions (MiPI), TERRA Teaching and Research Centre, Gembloux Agro-Bio Tech, University of Liège, 5030 Gembloux, Belgium; f.delvigne@uliege.be

**Keywords:** biological control, *Beauveria bassiana*, endophytic fungi, *Metarhizium anisopliae* s.l., non-target effects, *Spodoptera frugiperda*

## Abstract

The fall armyworm (FAW), *Spodoptera frugiperda*, is a highly polyphagous pest that has rapidly expanded across Africa, Asia, and Oceania, threatening food security and agricultural productivity. Reliance on synthetic insecticides for FAW management is increasingly challenged by insecticide resistance, environmental contamination, and adverse effects on non-target organisms and human health. This review synthesizes current knowledge on the potential of endophytic fungi as a sustainable strategy for FAW management. Following a systematic literature search, 59 original research articles published over the past 41 years were evaluated. These studies investigated 44 fungal species across 37 host crops, with *Zea mays* representing the most extensively studied system. The fungal species most frequently assessed were *Beauveria bassiana*, *Metarhizium anisopliae* sensu lato (s.l.), and *Epichloe coenophialum.* Overall, several fungal species successfully colonized plant tissues and reduced FAW performance through increased larval mortality, delayed development, and reduced feeding. These effects were associated with the production of bioactive secondary metabolites, induction of plant defense responses, and alteration of volatile organic compound emissions. Despite these promising findings, important knowledge gaps remain. Studies assessing effects on non-target organisms and natural enemies are scarce, and field validation remains limited. Research efforts are also geographically biased, with relatively few studies conducted in Africa despite the continent experiencing some of the highest FAW-related losses. Future research should prioritize field evaluations, multitrophic interaction studies, investigations in underrepresented regions, and socio-economic analyses to support farmer adoption. Addressing these gaps will be critical for determining the practical role of endophytic fungi in sustainable FAW management.

## 1. Introduction

The fall armyworm (FAW), *Spodoptera frugiperda* (J. E. Smith), is currently considered one of the world’s most destructive agricultural pests [1]. Native to tropical and subtropical regions in the Americas, this polyphagous Lepidopteran has rapidly expanded its range since 2016, reaching sub-Saharan Africa, Southeast Asia, Oceania, and, most recently, Europe [2,3,4]. Its invasive success is largely attributed to high fecundity, strong dispersal capacity, and an exceptionally broad host range [5]. Host plant records indicate that FAW can feed on at least 353 plant species across 76 families, with a marked preference for Poaceae (106 species), Asteraceae and Fabaceae [6]. In the absence of effective control strategies, maize, the principal host crop, can suffer yield losses of 10–58% [7,8,9], resulting in annual global economic losses estimated at several billion US dollars [10]. Current FAW management relies predominantly on synthetic chemical insecticides [11,12]. Although these compounds provide rapid suppression, their intensive use has led to widespread resistance across multiple insecticide classes, including pyrethroids, organophosphates, and diamides [13]. In addition, concerns regarding non-target effects, environmental persistence, and human health risks have reduced their sustainability and acceptability [14]. These constraints have driven a shift towards integrated pest management strategies that emphasize environmentally safer alternatives [15,16]. Among these, biological control using entomopathogenic fungi (EPF) has gained increasing attention due to their ability to infect and kill FAW larvae through articular penetration, without requiring ingestion, and their efficacy against non-feeding developmental stages [17]. These biological control agents help to protect several plant species from this pest [18]. Previous reports showed the ability of some Hypocrelean EPF of the genera *Beauveria* and *Metarhizium* can colonize plant tissues following artificial inoculation [19,20,21,22]. This endophytic lifestyle provides the fungi with a protected niche within plant tissues and may enhance plant resistance to herbivores while sometimes promoting plant growth [23,24]. Unlike conventional foliar applications, which depend on direct pest contact and repeated treatments, endophytic colonization can provide systemic and longer-lasting protection, potentially extending across multiple pest generations [25]. This characteristic is particularly advantageous for controlling FAW, as the larvae consume plant tissues colonized by endophytic fungi, thereby exposing them directly to fungal structures and/or secondary metabolites that may impair their development and survival [26].

Early studies demonstrated that grasses hosting systemic endophytes of the genus *Epichloe* (formerly *Neotyphodium*) experience reduced herbivore damage and enhanced fitness compared with endophyte-free plants [27,28]. These findings laid the conceptual foundation for understanding tripartite interactions between plants, fungi and insect herbivores [29]. However, the intentional use of EPF as endophytes for pest management is a relatively recent development that has gained considerable momentum over the past decade [30]. This growing interest reflects both the urgency created by the global spread of FAW [16] and advances in molecular tools enabling accurate detection and quantification of fungal endophytes [20].

Despite this progress, several critical knowledge gaps remain. First, the mechanisms by which endophytes affect FAW performance are not fully resolved and may involve antibiosis, feeding deterrence, plant-mediated defense induction, or a combination of these pathways. Second, although laboratory and greenhouse studies frequently report positive outcomes, field validations remain limited, and the ecological factors influencing colonization success and persistence are poorly understood [16]. Third, potential non-target effects on natural enemies, pollinators, and soil microbiota remain underexplored [31], despite their importance for environmental risk assessment and regulatory approval. Finally, there is a pronounced geographical imbalance in research efforts: most studies originate from the Americas and Asia, whereas Africa, despite bearing substantial FAW-related losses, remains underrepresented. This limits the development of locally adapted and accessible solutions, particularly for smallholder farming systems.

Therefore, a comprehensive synthesis of current knowledge on entomopathogenic endophytic fungi targeting FAW is timely. Such a synthesis is essential to clarify their efficacy and modes of action, identify consistent patterns of success and failure, and assess the robustness of existing evidence across experimental contexts. This review examines the diversity of fungal species reported as endophytes, host plants, and inoculation methods employed and their effects on different FAW life stages. It also evaluated emerging evidence on interactions with natural enemies, critically assesses methodologies used to confirm endophytic colonization and experimental design quality, and discusses technical, ecological, and socio-economic barriers to adoption. Ultimately, this synthesis aims to provide evidence-based guidance for future research and to support the integration of endophytic fungi into sustainable FAW management strategies worldwide.

## 2. Results

### 2.1. Spatio-Temporal Evolution of Endophytic Fungi Research on Fall Armyworm

**Figure 1:**
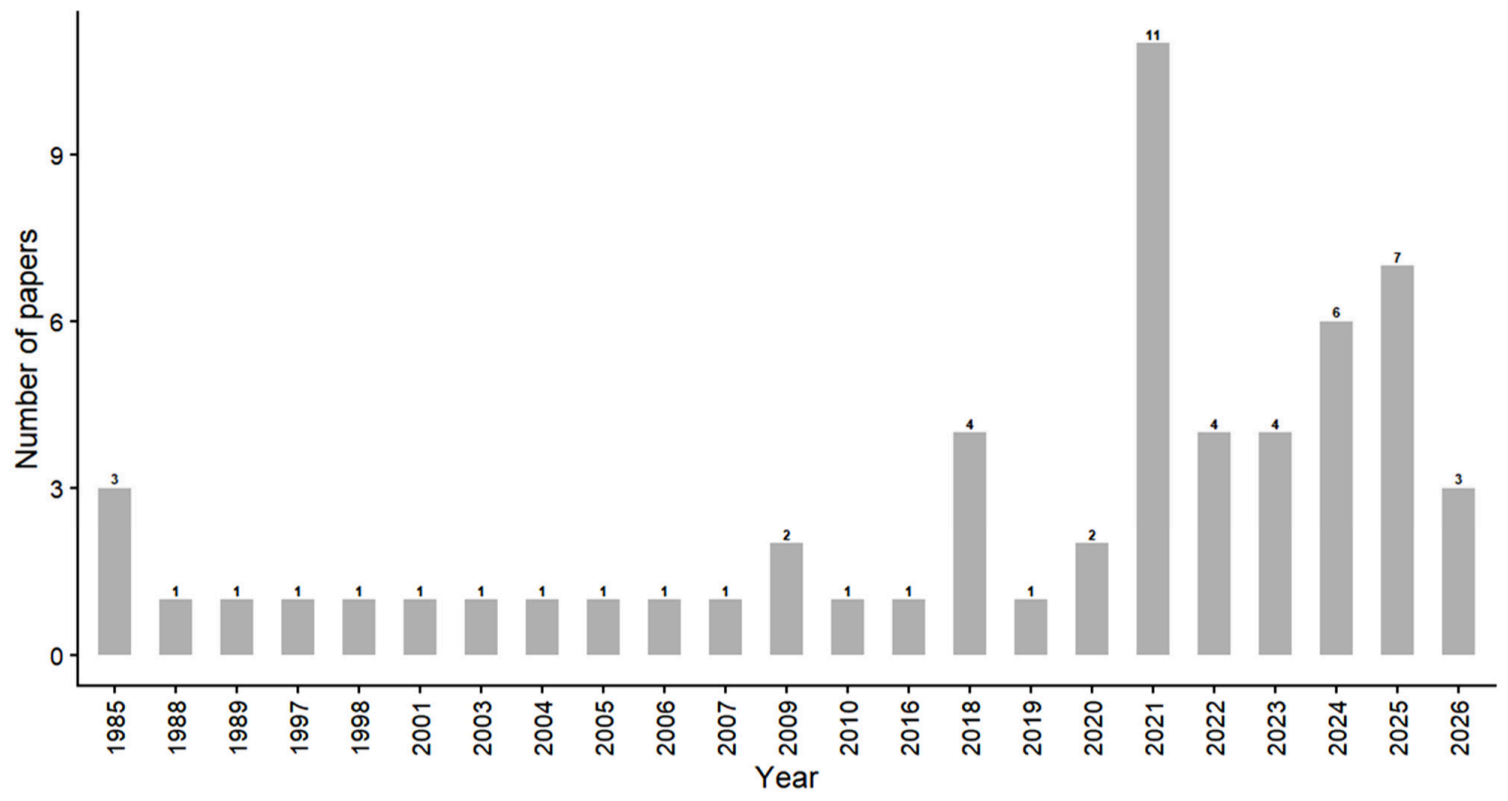
Number of publications on fungi use as endophytes against *Spodoptera frugiperda* per year. The numbers above the graphs represent the number of papers.

A total of 374 publications were initially identified through database searches. Following title and abstract screening, 85 papers were selected for full-text assessment. After applying the eligibility criteria, 59 studies were retained for final analysis. Research on the endophytic effects of fungi has been ongoing since 1985. However, a marked increase in publication output has occurred only in recent years. The highest annual number of publications was recorded in 2021, accounting for 18.6% of the included studies. Overall, 71.2% of the studies were published between 2018 and March 2026 (Figure 1), highlighting a strong recent growth in this research area.

**Figure 2:**
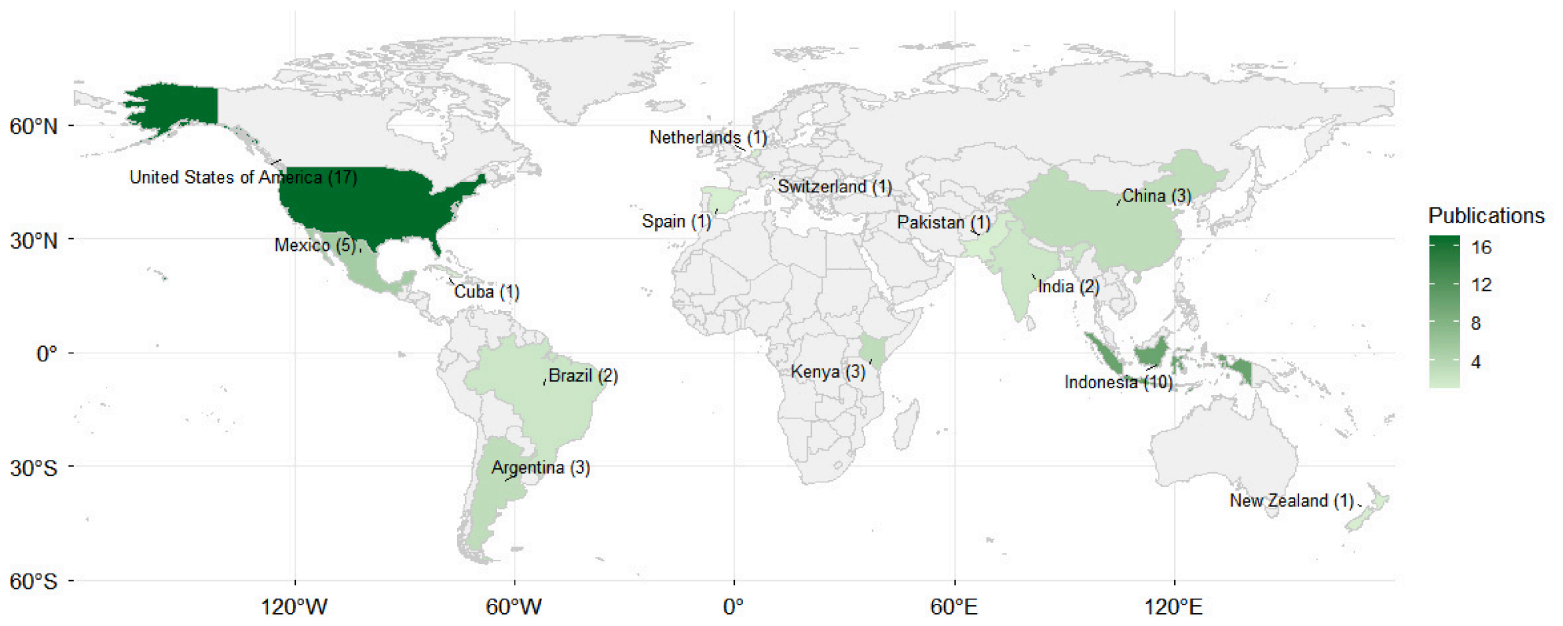
Number of papers on fungi used as endophytes against *Spodoptera frugiperda* per country in the world. The numbers following the country names indicate the number of publications from that country.

Research on the effect of endophytic fungi on FAW has been conducted in fourteen countries worldwide, with a clear dominance of studies from the Americas. This region accounts for 30 papers (50.8% of the total) (Figure 2). In Africa, research output remains limited, with Kenya contributing three publications. The Asian continent accounts for 22 publications, distributed across China, India, Indonesia, and Pakistan.

### 2.2. Fungal Diversity

**Figure 3:**
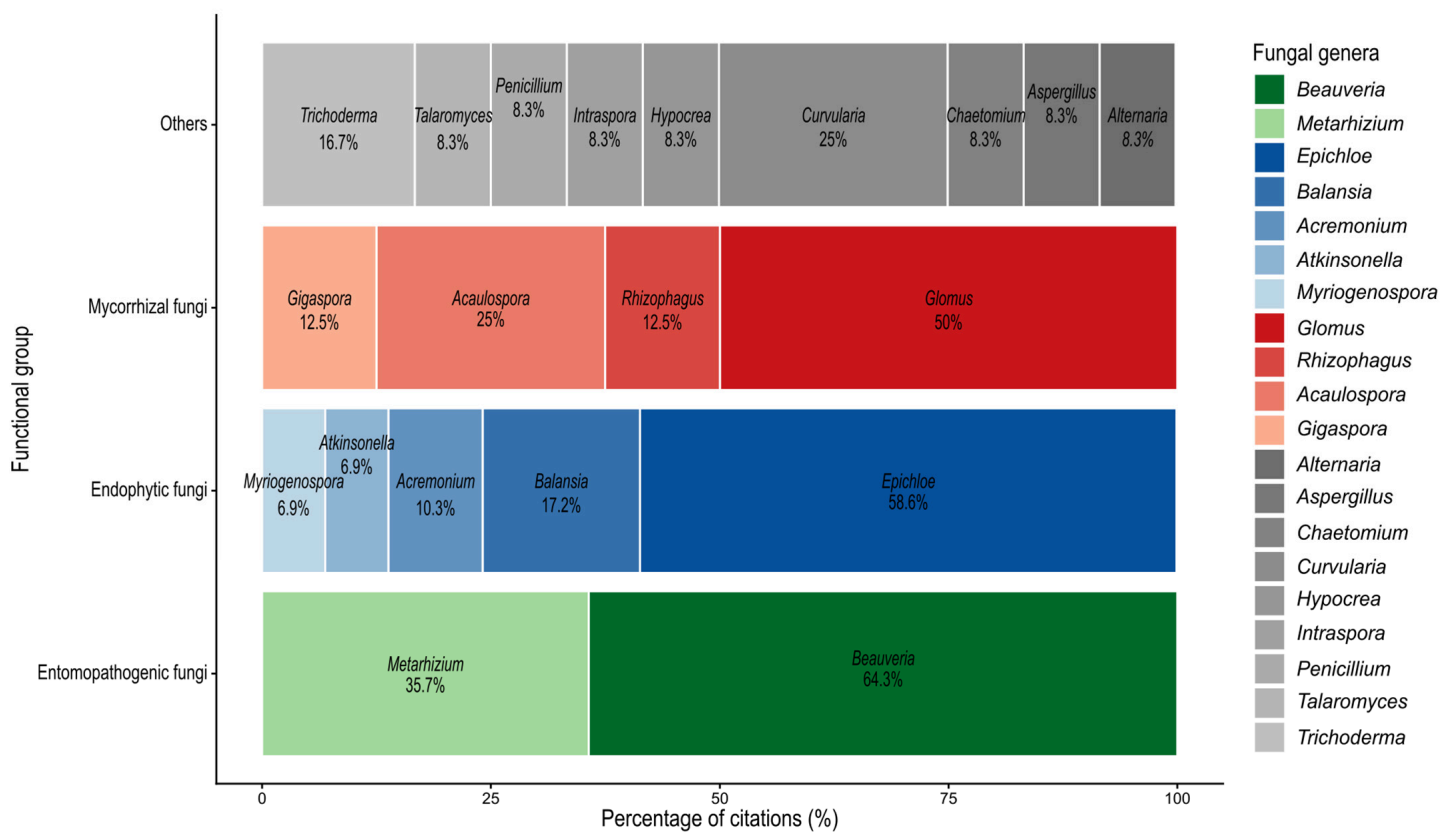
Diversity of fungi (*n* = 44 species) evaluated for their endophytic potential and effects on *Spodoptera frugiperda.* The studied fungi are categorized according to functional groups based on citation frequency. The percentage within each bar represents the contribution of each fungal genus to the corresponding functional group.

A total of 44 fungal species have been investigated for their ability to colonize plants and affect FAW or its natural enemies. These fungi were grouped into 3 functional categories: entomopathogenic fungi (EPF) [30,32], endophytic fungi [33,34], and mycorrhizal fungi [35]. At the study level, EPF were the most frequently investigated group, appearing in 71.9% of the selected articles, followed by mycorrhizal fungi (49.1%). In contrast, when considering species richness, endophytic fungi represented the most diverse group (43.9% of the fungal species), whereas entomopathogenic and mycorrhizal fungi accounted for 13.6% and 15.9% of species, respectively. At the genus level, *Beauveria* was the most frequently reported (43.1% of the publications), followed by *Metarhizium* (33.3%) and *Epichloe* (27.4%). EPF included only 2 genera: *Metarhizium* (35.7%) and *Beauveria* (64.3%). The genus *Metarhizium* was dominated by *Metarhizium anisopliae* s.l., and the genus *Beauveria* by *Beauveria bassiana* (Figure 3). Endophytes comprised 5 genera, dominated by the genus *Epichloe* (58.6%), with *Epichloe coenophialum* being the most studied. Mycorrhizal fungi were grouped into 4 genera, with the genus *Glomus* being the most studied (50%).

Fungi were isolated from multiple sources, including soil [32,36,37,38], plant tissues [39,40], and insect cadavers [37,39]. The most studied fungal species was *B. bassiana*, reported in 25 studies, followed by *M. anisopliae* s.l., included in 11 studies. *E. coenophialum* ranked third and was primarily studied in the Americas, where it represents a foundational model in plant-fungus mutualism research. Overall, entomopathogenic and endophytic fungi have received substantially more attention than mycorrhizal taxa, highlighting a taxonomic bias and indicating potential avenues for future research.

### 2.3. Crop Diversity

**Figure 4:**
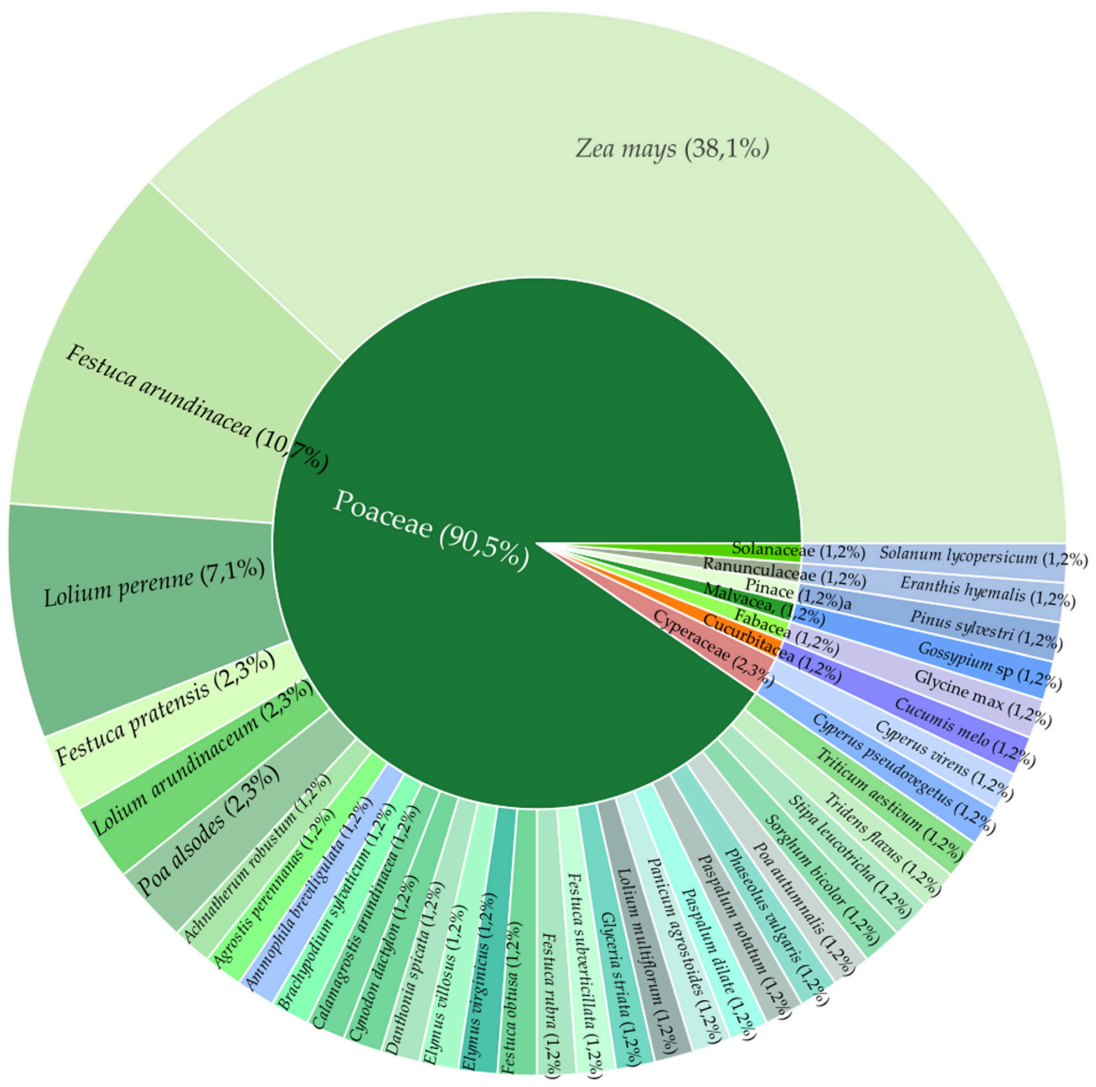
Host plant species (*n* = 37) that were used in the studies to evaluate the colonization capacity and efficacy of endophytic fungi in controlling *Spodoptera frugiperda*. The plant families used are shown in the center, and the species studied are shown on the periphery. The percentages below the names represent the frequency with which the species was cited relative to all species studied.

The effects of endophytic fungi on FAW and/or its natural enemies were evaluated across a wide range of host plants, primarily within the Poaceae family, including genera such as *Zea*, *Lollium*, and *Festuca*. Nevertheless, other plant families have been studied, including the Cyperaceae, Curculiaceae, Fabaceae, Malvaceae, Pinaceae, Ranunculaceae and Solanaceae. However, these families have not been studied as extensively as the Poaceae (Figure 4). The main objective of these studies was to assess the ability of endophytic fungi to colonize different plant species and to determine their impact on the pest and its natural enemies. In total, 37 crop species were investigated, with grass representing the majority (90%) of hosts. *Zea mays* was the most frequently used crop, appearing in 54.2% of the selected studies, followed by *Festuca arundinacea* (20.3%) and *Lolium perenne* (11.9%). Other crops, including *Sorghum bicolor*, *Gossypium* sp., and *Solanum lycopersicum*, were also evaluated, although less frequently, indicating interest in extending this approach beyond major cereal hosts.

### 2.4. Inoculation Methods for Endophytic Fungi

**Figure 5:**
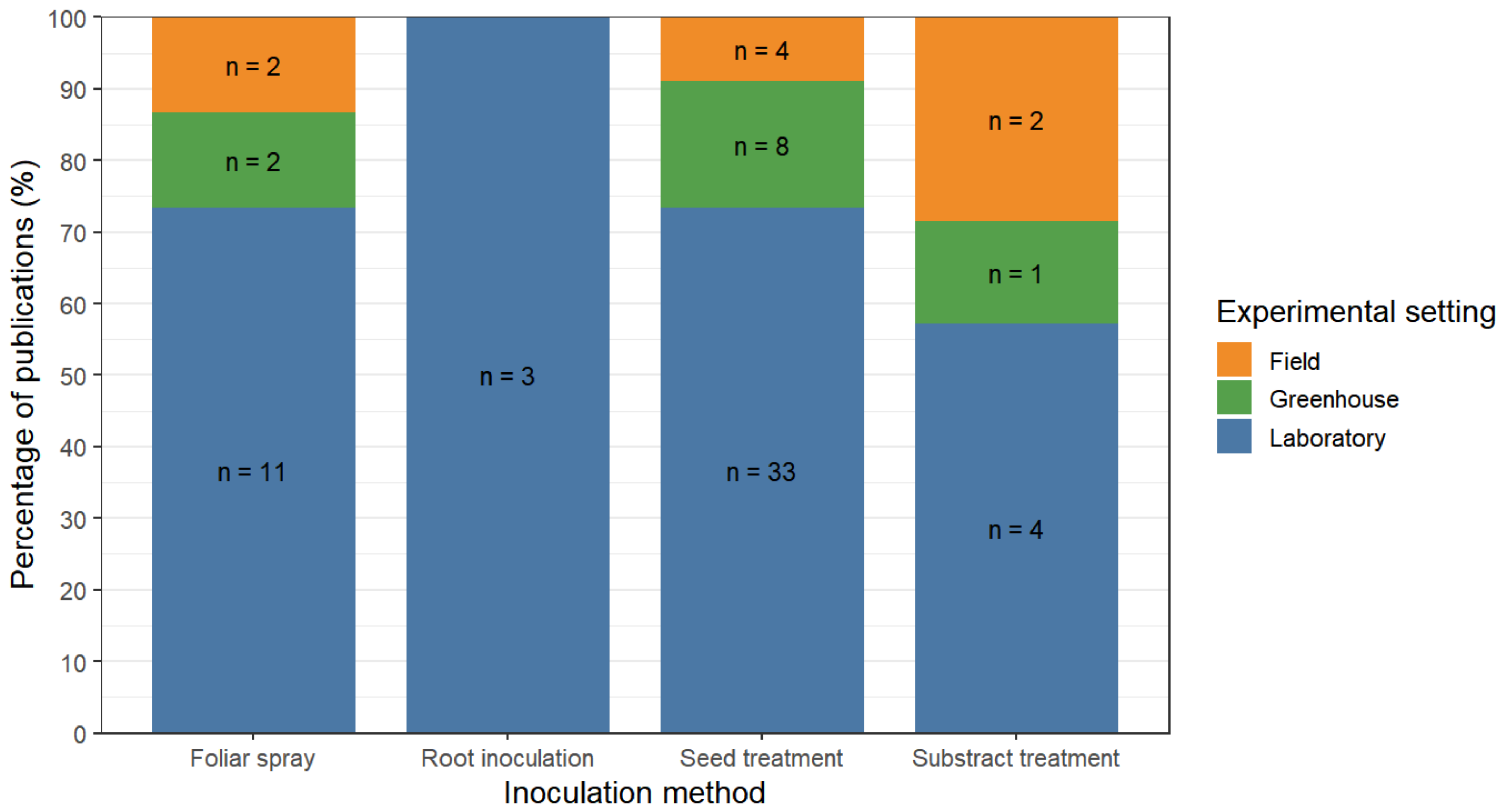
Inoculation methods used to study the effects of endophytic fungi on *Spodoptera frugiperda* and/or its natural enemies. Colors indicate the experimental setting (field, greenhouse, or laboratory), and numbers represent the number of publications for each inoculation method and setting.

The studies were conducted in laboratories (79.7%) and greenhouses (22%), with very few field studies (0.1%) (Figure 5). Inoculation approaches include seed treatments (e.g., soaking, immersion, or coating), foliar applications (e.g., spraying or leaf inoculation), root dipping, and soil or substrate inoculation. Seed treatment was the most commonly used inoculation method, followed by foliar application. Root inoculation was performed only in the laboratory. Endophytic colonization has been demonstrated for several fungal species. Fungal endophytes and entomopathogens are introduced into plants using a range of inoculation techniques, depending on the fungal group, host plant, and experimental objectives. Reported host plants capable of supporting endophytic colonization include *Zea mays* [24,36,41,42,43,44,45]; *Festuca arundinacea* [28,46,47]; *Lolium* sp. [38,46]; *Sorghum bicolor* [48]; *Gossypium* sp.; *Cucumis melo* [39]; *Phaseolus vulgaris* [49]; *Poa alsodes* [50]; *Solanum lycopersicum* [51]; *Cyperus virens*; and *Cyperus pseudovegetus* [27]. In most studies, conidia, spores, or blastospores are used as inoculum sources, particularly for entomopathogenic taxa. In contrast, studies on naturally occurring endophytes frequently rely on plant material or seeds already colonized by fungi, particularly in grasses, such as *Lolium* [52,53,54] and *Festuca* species [47,55,56,57,58,59]. Different fungal groups exhibit distinct colonization patterns within host plants. Mycorrhizal fungi are generally restricted to the root system, whereas endophytic and entomopathogenic fungi may colonize multiple plant tissues, including roots, stems, and leaves [24,33,41,43,44,60,61,62]. Colonization success and intensity depend on several interacting factors, including plant organ, fungal species, inoculation method, inoculum type, and host-fungus compatibility [24,41,43,44,62,63]. Following establishment within plant tissues, endophytic fungi may require a period of adaptation before stable colonization is achieved. Once established, they may exert multiple effects, including plant growth promotion in some cases and antagonistic effects on insect pests such as FAW [24,43,49,64].

In general, *B. bassiana, M. anisopliae* s.l., and *Trichoderma* sp. have been observed to exhibit plant colonization rates of up to 100% (a rate that varies according to the plant organ concerned). Furthermore, [65] demonstrated that a high colonization rate of *B. bassiana* can be sustained until harvest, particularly under conditions of elevated CO_2_ levels.

### 2.5. Effects of Endophytic Fungi on Fall Armyworm

Different developmental stages of FAW were used across the reviewed studies, with the larval stage being the most frequently assessed (91.5% of papers). In these experiments, larvae were typically fed leaves colonized by endophytic fungi. Studies examining the effects of fungi on FAW pupae and adults were generally conducted alongside studies on larvae, accounting for 20.3% and 13% of studies, respectively. The role of these fungi in FAW management includes reducing insect development and larval survival, prolonging larval lifespan, decreasing larval and pupae weight, deterring larval feeding on colonized leaves, enhancing plant defense enzyme activity and hormone concentration levels, and reducing adult fertility. Indeed, the hatching of eggs laid by adults that originated from larvae that consumed leaves colonized by fungi may be delayed, and the duration of the larval development cycle may be prolonged [66]. Furthermore, the emergence of adults from the pupae of these larvae may be delayed [37,40,63]. Several studies have demonstrated that larvae fed on infected leaves have reduced weight, resulting in reduced pupal mass [51,60]. The number of adults emerging from pupae derived from larvae that have consumed leaves colonized by certain fungi, as well as the lifespan of the resulting adults, may be reduced [37,66].

Certain fungi, including *B. bassiana* and *M. anisopliae* s.l., were reported to cause larval mortality exceeding 50%, reduce adult emergence by approximately 70%, and decrease fecundity by around 20% [37,41,63,67]. Similar effects have also been reported for non-entomopathogenic endophytes. For example, fungal endophytes associated with grasses have been shown to reduce larval survival and weight following consumption of colonized plant tissues [28,53,68]. Behavioral responses were also reported. Most studies indicated that FAW larvae preferentially feed on non-colonized leaves [46,53], although some studies suggested that larvae may not reliably distinguish between colonized and non-colonized tissues [42,69]. Reproductive effects were also observed in adults. In particular, fungal exposure has been associated with altered sex ratios, reduced mating success, and decreased female fertility [50]. To enhance the fungi effect on larvae, some researchers have used them (*B. bassiana* and *Alternaria alternata*) in combination with CO_2_ [65]. However, not all studies reported significant effects, as some fungal strains showed no measurable impact on larval performance following consumption of colonized leaves [38,42] (Table 1).

### 2.6. Non-Target Impacts on Natural Enemies

Only seven studies have examined the effects of endophytic fungi on FAW natural enemies, indicating that this multitrophic aspect remains largely unexplored. The main natural enemies under scrutiny in this context include *Telenomus remus* [78], *Euplectrus comstockii* [45,56], *E. plathypena* [87], *Steinernema carpocapsae* [54], *Campoletis sonorensis* [85], and a multitude of predators [77]. The authors demonstrated that fungi, after plant colonization, have the capacity to influence the interactions between the host, the pest, and the natural enemies [57] by inducing changes in host characteristics. For instance, the performance and survival of *E. comstockii*, a larval parasitoid of FAW, were reduced by *Epichloe* fungi [46]. As demonstrated in another study [57], the effect of the fungus on the parasitoid is contingent upon the strain of fungus utilized. The survival rate of *E. comstockii* was reduced by *E*. *coenophialum* AR542 and CS isolates, but not by the AR502 isolate. Another fungus, *Acremonium coenophialum*, had a negative impact on the hymenopterans *Euplectrus comstockii* and *Euplectrus plathypenae* after colonizing *Festuca arundinacea*, although the authors acknowledge that this impact was moderate [57]. As demonstrated by [54], *Epichloe lolii* reduced the susceptibility of FAW larvae to *Steinernema carpocapsae*, an entomopathogenic nematode that infects and destroys insects. Experiments conducted with the bacterium *Xenorhabdus nematophila*, which is secreted by the nematode, demonstrated effects on the larvae. Larvae that had been fed on corn leaves with roots colonized by *T. atroviride* by *Campoletis sonorensis* exhibited a parasitism rate that was 50% higher than those from uninfected plants [85]. Egg parasitism by *Telenomus remus* was not affected by *B. bassiana* endophytic colonization [78]. The impact of this fungus on the diversity and abundance of predatory arthropods in the field was not significant when it was used as an endophyte [77]. Overall, the limited number of studies and the contrasting outcomes reported indicate that the effects of endophytic fungi on FAW natural enemies are highly context-dependent and remain insufficiently understood.

## 3. Discussion

The expansion of research on endophytic fungi in response to the recent global spread of FAW reflects the urgent need for sustainable pest management strategies following the emergence of this invasive pest. However, it also exposes critical geographical imbalances and knowledge gaps and highlights the complexity of tri-trophic interactions that must be addressed to translate laboratory findings into practical solutions. The surge in publications since 2020 reflects the scientific community’s response to the rapid intercontinental spread of the FAW following its introduction into West and Central Africa in 2016 [2]. This crisis-driven acceleration is characteristic of invasive pest research, where urgent agricultural threats stimulate the exploration of sustainable alternatives [17]. Despite this increase in research activity, the geographical distribution of studies highlights a persistent imbalance that may limit the global applicability of current findings. The Americas dominate the literature, likely reflecting both the native range of FAW and a long-standing research tradition in plant-endophyte interactions. Recent studies have further expanded this field by demonstrating the potential of EPF as phytoendophytes [26]. Asia follows, consistent with the eastward spread of FAW [88]. In contrast, Africa, despite experiencing severe economic impacts from FAW, has contributed only three studies, all conducted in Kenya. This mismatch between research effort and regional need is concerning, as African agroecosystems are characterized by smallholder farming systems, intercropping practices, and environmental conditions that differ substantially from those in regions where most experiments have been conducted. The majority of rural farming households in sub-Saharan Africa cultivate less than 2 hectares of land [89] and often have limited financial resources. These smallholder systems are frequently based on mixed cropping, where interactions among plant species may influence the establishment and activity of introduced endophytic fungi, potentially leading to synergistic or antagonistic effects [90,91]. Consequently, the efficacy observed in maize monoculture may not directly translate to more complex intercropping systems. In addition, farmers often prioritize pest management strategies that are affordable, reliable, and supported by accessible technical advice [92]. The adoption of fungal endophyte-based technologies may therefore depend on demonstrating their economic benefits and reliability under local farming conditions. Environmental factors, including ultraviolet radiation, low humidity, and drought conditions, may also influence fungal persistence and efficacy [30]. These factors, combined with limited access to irrigation and limited financial resources, highlight the need for locally generated data on fungal performance against endemic FAW populations and their compatibility with regional farming practices [17]. Strengthening research capacity, North–South collaborations, and targeted investment in biocontrol research will be essential to facilitate the adoption of fungal endophytes in sub-Saharan Africa.

The taxonomic diversity captured in this review (44 species across entomopathogenic, classical endophytic, and mycorrhizal functional groups) indicates that endophytism is a widespread ecological strategy rather than a rare trait [93]. The dominance of *B. bassiana* and *M. anisopliae* s.l. is consistent with their established use as commercial biopesticides and their broad ecological plasticity. Continued interest in classical endophytes such as *Epichloë* reflects their importance as model systems for plant-fungus mutualisms [83].

The authors used several inoculation methods, with seed treatment being the most prevalent. In terms of the ability to colonize host plants, this method also yielded good results. This approach could facilitate the large-scale use of endophytes for field applications. Among the inoculated crops, maize was the most used. This could be related to the bioecology and feeding preferences of the pest, as maize is one of the main host crops [94,95]. *S. frugiperda* is also known to have two strains: strain C (Corn), which preferentially colonizes maize, and strain R (Rice), which feeds on rice and other small grasses [96,97]. However, rice is not among the crops studied. Future studies could include rice and help elucidate the behavior of endophytic fungi on this crop as well as their potential impact on the pest. Although most *S. frugiperda* populations, particularly in invaded areas, have been reported from maize and sorghum [98,99,100], rice and other crops may become alternative hosts where preferred crops are unavailable. The potential performance of endophytic fungi in the R-strain system remains largely unexplored. Nevertheless, rice is known to harbor diverse endophytic fungal communities [101] and introduced EPF such as *B. bassiana* and *M. anisopliae* s.l. have successfully colonized rice tissues using inoculation methods similar to those used for other cereals [102]. For instance, *Lecanicillium saksenae* persisted for up to 90 days after seed inoculation and promoted plant growth [103]. However, rice defense mechanisms differ from those of maize, which may influence plant-fungus-insect interactions, including volatile organic compound (VOC)-mediated responses affecting R-strain larvae [104]. The R-strain may also exhibit differential susceptibility to fungal endophytes compared with the C-strain, given reported differences in host range and detoxification capacity [105,106]. Furthermore, the flooded or intermittently flooded conditions characteristic of rice agroecosystems may affect fungal establishment and persistence. Nevertheless, root endophytes such as *Piriformospora indica* have successfully colonized rice and induced tolerance to root herbivory through gibberellin-mediated modulation of jasmonate signaling, suggesting that rice-associated endophytes may still establish beneficial plant interactions under flooded or intermittently flooded conditions [104]. Understanding the behavior of endophytic fungi in rice systems could offer several advantages for controlling *S. frugiperda*. After colonizing rice plants, endophytic fungi may enhance plant growth and resistance to biotic and abiotic stresses [107,108]. They may also reduce *S. frugiperda* damage by slowing insect development [109,110], decreasing larval survival and oviposition [111], and reducing feeding on rice leaves [30,110].

A key finding of this synthesis is the high degree of intra-specific variability in both colonization success and pest impact. The contrasting performance of different *Metarhizium* isolates to establish in maize [44] illustrates that species-level identification alone is insufficient to predict biocontrol outcomes. Instead, efficacy depends on a specific molecular interaction between fungal strain and host plant [24]. This interaction likely involves immune evasion, nutrient acquisition within the apoplast, and strain-specific secondary metabolite production. The ability of fungi to acquire and utilize plant-derived nutrients is an important factor influencing successful plant colonization. A study investigating the diversity of *M*. *robertsii* strains collected from the rhizosphere revealed substantial variation in fungal phenotypes among strains [112], highlighting that rhizosphere colonization mechanisms may be strain-dependent [113]. A further study [114] identified mutants of the *Metarhizium* raffinose transporter (Mrt) gene in *M. robertsii*, which encodes a sucrose and galactoside transporter. These findings indicate that this transporter contributes to fungal growth within plants without affecting insect virulence. Other studies have shown that MAD1 and MAD2 proteins play important roles in *M*. *anisopliae* s.l. virulence, root adhesion, and colonization [115]. Plant–microorganism associations are accompanied by transient immune responses in host plants [116], which microorganisms must modulate to establish successful interactions. Colonization by *M. brunneum* and *B. bassiana* has been associated with downregulation of plant immunity-related genes [117]. Fungal colonization can also influence reactive oxygen species (ROS) in host plants, as observed in *M. brunneum, M. robertsii* and *B. bassiana* [117,118]. Several studies have identified secondary metabolites produced by different fungal strains and their contribution to fungal effectiveness [119]. For example, the cyclopeptides destruxin in *M. robertsii*, beauvericin and bassianolide, and the benzoquinone oosporein in *B. bassiana* contribute to fungal virulence, with their biosynthesis involving NRPS or PKS genes [120,121,122]. However, the contribution of individual metabolites may vary among strains, as demonstrated by the inactivation of the NRPS gene encoding serinocyclin synthase (DMaNPS1) in *M. anisopliae*, which resulted in a strain that remained virulent against lepidopteran and coleopteran hosts [119].

Consequently, biocontrol development is unlikely to succeed under a “one-size-fits-all” approach and may instead require region- or crop-specific elite strains. Future research should prioritize genomic and metabolomic approaches to identify markers associated with successful endophytic colonization and pest suppression.

The studies reviewed indicate that endophytic fungi protect plants through a multi-layered strategy extending beyond direct infection. In grass-associated *Epichloe* endophytes, alkaloids such as ergovaline, peramine, and ergot derivatives contribute to reduced larval performance and survival [59,83]. However, indirect mechanisms are equally important. The priming of plant defense pathways, evidenced by increased activity of enzymes such as polyphenol oxidases, phenylalanine ammonia lyases, and lipoxygenases [61], suggests activation of plant defense responses and indicates that fungal colonization may enhance the plant’s immune capacity. This aligns with the concept of defense priming, whereby endophytes induce a heightened defensive state in host plants [52]. In addition, VOCs contribute to plant-insect-natural enemy interactions. Feeding deterrence on colonized plants [78] and parasitoid attraction observed in *Trichoderma*-inoculated systems [85] suggest that endophytic fungi can influence both bottom-up (plant quality) and top-down (natural enemy attraction) ecological processes. This multi-mechanistic action may reduce the likelihood of resistance development by complicating adaptive responses in FAW, an insect with strong detoxification capabilities [123], thereby making resistance evolution more difficult. The reported ability of mycorrhizal fungi to enhance insecticide uptake and efficacy [45] further illustrates the diverse roles the fungi may play in pest management systems.

The scarcity of studies examining interactions with natural enemies (only seven) represents one of the most significant knowledge gaps identified in this review. The available data reveal a complex pattern that prevents broad generalizations. While some parasitoids appear to be affected by the presence of endophytes [45,84], others, including *T. remus*, and certain generalist predators [77,78], are not affected by the presence of the fungus within the plant. Negative effects have been associated with alkaloid-producing endophytes in grasses, where reductions in parasitoid performance [46] suggest that plant-associated defensive compounds may move through the food chain and affect higher trophic levels, raising potential concerns for conservation biological control. In contrast, positive effects have been reported in systems involving *Trichoderma*, where volatile organic compounds (VOCs) enhanced parasitoid attraction [85], indicating that endophytes can also strengthen top-down biological control. Overall, these findings highlight that endophytic fungi may either disrupt or enhance biological control depending on the fungal species, strain, and ecological context, which has important implications for integrated pest management. An effective fungus against FAW under laboratory conditions may therefore produce unintended effects on beneficial organisms under field conditions.

The predominance of laboratory and greenhouse experiments highlights a key translational limitation. While controlled experiments are essential for mechanistic understanding, they do not fully capture field conditions, where variability, soil microbiota, and climatic factors influence fungal establishment and persistence [47,58]. Moreover, most studies focus on insect mortality or development rather than agronomic outcomes such as yield. Future research should therefore focus more on field studies to ensure that the use of fungi as endophytes does not compromise the ecological role of natural enemies [124]. The barriers to adoption identified in this review, including inconsistent field performance, high formulation costs, regulatory complexity, and limited farmer awareness, are interconnected. It is reasonable to hypothesize that a farmer accustomed to the rapid, visible knockdown of a synthetic pesticide is unlikely to adopt a biological product with subtle, long-term effects unless its economic benefits are clearly demonstrated and communicated. Farmers are unlikely to adopt biocontrol products without clear and consistent evidence of economic benefit. In addition, strain specificity complicates product standardization and scalability. Addressing these challenges requires not only agronomic research but also socio-economic studies, participatory approaches, and strengthened extension systems.

Overall, the evidence indicates that endophytic fungi represent a multifunctional but highly context-dependent tool for FAW management. Field validation is urgently required to assess fungal persistence, pest suppression, crop yield effects, and impacts on non-target organisms across agroecological zones. Research must also prioritize tri-trophic interactions to ensure compatibility with other biological control agents and robust IPM integration. Advances in genomics and metabolomics offer promising avenues for identifying high-performance strains, while socio-economic studies are essential for understanding adoption constraints and farmer perceptions. Collectively, these efforts provide a roadmap for translating the potential of endophytic fungi into practical and sustainable FAW management strategies.

## 4. Methodology

To identify scientific literature addressing endophytic EPF and their effects on FAW and/or its natural enemies, a comprehensive literature search was conducted using combinations of the following keywords: “*Spodoptera frugiperda*” or “Fall armyworm” or “chenille légionnaire d’automne” AND “endophytic fungi” or “endophytic entomopathogenic fungi” AND “natural enemies” or “parasitoids” or “parasites” or “predators”. Searches were performed in major scientific databases and search engines, including Scopus, Springer Nature, ScienceDirect, and Google Scholar. Retrieved publications were initially screened based on their titles and abstract. Studies were included when they met the following criteria: (i) they were original peer-reviewed research articles published in scientific journals; (ii) they investigated endophytic fungi or entomopathogenic fungi used as endophytes in relation to FAW and/or its natural enemies; and (iii) they evaluated the effects of fungal colonization on FAW (eggs, larvae, pupae, or adults) or on its natural enemies, including predators and parasitoids. Review articles, meta-analyses, conference abstracts, and other non-primary research documents were excluded. The studies were examined in detail to extract the following information: study objectives, publication date, country of study, fungal species investigated and their origin, host crops used, inoculation methods, degree of host plant colonization, biological material evaluated, effects of the fungus on the pest and/or its natural enemies, experimental setting (laboratory, greenhouse, or field), and main findings.

Effects on FAW were classified as positive, neutral, or negative from the perspective of pest management. A positive effect corresponded to any impact of the fungus that adversely affected at least one developmental stage of the pest. A neutral effect indicated no measurable impact on any developmental stage. A negative effect referred to situations in which fungal colonization increased pest performance or reduced the effectiveness of natural enemies. For natural enemies, effects were also classified as positive, neutral, or negative. A positive effect corresponded to increased parasitism or predation in the presence of the endophyte, thereby contributing to pest suppression. A negative effect corresponded to reduced parasitism or predation, whereas a neutral effect indicated no detectable influence. When multiple fungal species were evaluated within a single study, only those demonstrating successful endophytic colonization were retained for analysis. Their reported effects, whether positive, neutral, or negative, were recorded. Fungal taxa were subsequently grouped according to their functional categories [34,35,125]. Species formerly assigned to the genus *Neotyphodium* were classified within the genus *Epichloe* following the taxonomic revision of [126]. Data compilation, statistical analyses, and graphical visualizations were performed using R software version 4.5.3 [127]. This review was conducted in accordance with the 2020 PRISMA (Preferred Reporting Items for Systematic Reviews and Meta-Analyses) guidelines [128] (Supplementary Materials).

## 5. Conclusions

This review highlights the potential of endophytic fungi as a sustainable strategy for managing FAW. Across the studies examined, they were shown to affect multiple aspects of FAW biology, including development, survival, feeding behavior, and reproduction, through direct and indirect mechanisms. However, their efficacy is highly variable and depends on fungal species and strain, host plant, inoculation method, and environmental conditions. Despite increasing research interest, important gaps remain. Most studies have been conducted under laboratory or greenhouse conditions, with limited field validation. In addition, information on non-target effects on natural enemies, pollinators, and other beneficial organisms remains scarce. Marked geographical disparities also exist, particularly in regions most affected by FAW, such as sub-Saharan Africa. Future research should prioritize field validation across diverse agroecological conditions, improve understanding of fungal colonization and pest suppression mechanisms, and assess compatibility with integrated pest management systems. Advances in genomics, transcriptomics, metabolomics, and microbiome research may further support the identification of fungal traits linked to successful colonization and pest control, enabling improved strain selection and more reliable field performance. Overall, these developments will be essential for defining the practical role of endophytic fungi in sustainable FAW management and supporting their effective adoption in agricultural systems.

## Figures and Tables

**Table 1:** Fungi investigated for their ability to colonize plants and their effect on *Spodoptera frugiperda*. Effects on *S. frugiperda* are classified as positive (+), negative (−), or neutral (0). Fungi are grouped according to their functional groups.

Fungal Groups	Fungal Species	Effect on *Spodoptera frugiperda*			Number of Papers	References
		(+)	(0)	(−)		
Entomopathogenic fungi	*Beauveria bassiana* and *Beauveria* sp.	♦			27	[32,37,39,40,41,43,44,48,49,51,60,62,63,64,65,66,70,71,72,73,74,75,76,77,78,79,80]
	*Metarhizium anisopliae* s.l.	♦			11	[39,41,51,60,63,67,71,72,74,79,81]
	*Metarhizium rileyi*	♦			1	[43]
	*Metharizium robertsii*	♦	♦		2	[81]
	*Metharizium humberi*	♦			1	[81]
Endophytic fungi	*Epichlo* *e coenophialum*	♦		♦	6	[46,56,57,58,59,69]
	*Epichlo* *e* *lolii*	♦		♦	2	[54,69]
	*Acremonium lolii*	♦			1	[82]
	*Acremonium* *coenophialum*	♦		♦	2	[56,82]
	*Atkinsonella hypoxylon*, *Myriogenospora atramentosa*	♦			2	[28,82]
	*Epichloe* sp.	♦			9	[27,28,47,50,55,59,68,83,84]
	*Balansia* sp.	♦			4	[27,28,82,83]
Mycorrhizal fungi	*Acaulospora delicata* and *Acaulospora* spp.		♦	♦	2	[36,38]
	*Glomus intraradices*, and *Glomus mossea*, *Glomus* spp.,	♦		♦	3	[36,45,61]
	*Gigaspora* spp., and *Intraspora* spp.			♦	1	[36]
	*Rhizophagus irregularis*	♦			1	[80]
Other fungi	*Penicillium citrinum*		♦		1	[71]
	*Trichoderma atroviride* et *Trichoderma* sp.	♦			2	[24,85]
	*Hypocrea lixii*	♦			1	[49]
	*Talaromyces verruculosus*	♦			1	[86]
	*Aspergillus* sp.; *Chaetomium* sp. and *Curvularia* sp.	♦			1	[40]
	*Alternaria alternata*	♦			1	[65]
	*C. lunata*	♦			2	[39,72]

## Data Availability

No new data were generated or analyzed in this study. This article is a review of previously published research; all data discussed are available in the original publications cited in the reference list.

## Supplementary Materials

The following supporting information can be downloaded at: https://www.mdpi.com/article/10.3390/plants15152375/s1, Prisma 2020 Checklist.
